# Supplementary material for: Noncanonical functions of Ku may underlie essentiality in human cells
Source: Sci Rep. 2023 Jul 27;13:12162. doi: 10.1038/s41598-023-39166-7 (PMC10374653; doi:10.1038/s41598-023-39166-7)
Supplement: Supplementary file 1 — Supplementary Figures. [file 41598_2023_39166_MOESM1_ESM.pdf]

Noncanonical functions of Ku may underlie essentiality in human cells

Rachel D. Kelly[1,2], Gursimran Parmar[1,2], Laila Bayat[1,2], Matthew E.R. Maitland[1,2], Gilles A. Lajoie[1], David R. Edgell[1], Caroline Schild-Poulter[1,2]

Supplementary Information

Figure Legends

Figure S1. Targeting Three Exon/Intron Junctions for CRISPR editing

Three gRNAs were designed to target the Exon/Intron junctions of Exons 7, 6, and 12 respectively. SaCas9 or TevCas9 endonucleases were used to induce cleavage at the target sites. Expected cut sites are indicated by red lines in the sequence.

Figure S2. Western blots and quantification of Dox depletion curves for Sa11 and TI Ku70 knockout clones

a. Western blot of Dox depletion curve for the Sa11 Ku70 knockout clone on the indicated days. Whole cell extracts from Sa11 cells cultured in presence (+) or absence (-) of Dox and were analyzed by western blot with the indicated antibodies. Quantification of Ku70 and Ku80 relative to alpha-tubulin plotted as the mean of 3 biological replicates with error bars reporting +/- SEM. * indicates Ku70 or Ku80 is significantly changed compared to Day 1 Dox ($p<0.05$). **b.** Western blot of Dox depletion curve for the TI Ku70 knockout clone on the indicated days. Whole cell extracts from TI cells cultured in presence (+) or absence (-) of Dox and were analyzed by western blot with the indicated antibodies. Quantification of Ku70 and Ku80 relative to alpha-tubulin plotted as the mean of 3 biological replicates with error bars reporting +/- SEM. * indicates Ku70 or Ku80 is significantly changed compared to Day 1 Dox ($p<0.05$).

Figure S3. Average γH2AX foci accumulation does not change significantly following Dox withdrawal compared to IR treated cells

a. The average number of γH2AX foci/nucleus in Ku70 knockout cells on the days indicated in media containing Dox or following Dox withdrawal (no Dox) (N=4). TREx-293 Ku70-HA cells treated with 2 Gy of ionizing radiation (IR) act as a positive control. For each replicate, >50 nuclei were analyzed per condition. TREx-293 Ku70-HA cells treated with 2 Gy of ionizing radiation are significantly different from untreated TREx-293 Ku70-HA cells and Ku70 knockout cells as denoted by * symbol, but average foci accumulation in knockout cells is not significantly different from unedited TREx-293 Ku70-HA cells as denoted by ns (Ordinary One-Way ANOVA, multiple comparisons, $p<0.001$).

Figure S4. Volcano plots of altered proteins following Ku70-HA withdrawal

Proteins found to be decreased or increased significantly on **a.** Day 4, **b.** Day 6, and **c.** Day 7 post Dox withdrawal (Fold-change ≥ 1.5; p-value ≤ 0.05). The gray line bisecting the y-axis denotes a p-value of 0.05. Following Ku withdrawal, proteins with a fold-change of 1.5 or greater compared to growth-matched controls for each day are denoted by red points on the volcano plot. Proteins that are decreased compared to controls have a negative log(Fold Change) and proteins that are increased have a positive log(Fold Change).

Figure S5. Western blot validation of proteomic data for EIF3B, MYO6, and PDCD4 candidates in alternate Ku70 knockout clone, Sa11

a. Western blot validation of proteomic analysis results for 3 candidate proteins at the days listed post Dox withdrawal for the Sa11 Ku70 knockout clone. All samples are from

one experiment, but the top and bottom panels are from 2 different western blots. **b.** Quantification of MYO6, EIF3B, and PDCD4 protein levels relative to alpha-tubulin for (N=3) western blots. +/- indicates the presence or absence of Dox from cell media. Day post Dox withdrawal are indicated.

Figure S6. Unprocessed western blots

Figure S7. Unprocessed Southern blot from Telomere Restriction Fragment Analysis

S1 Data. Sanger sequencing data and DECODR analysis confirmation of Ku70 editing at target sites 1 and 2 for Sa11, SB, and TI Ku70 knockout clones

S2 Data. List of proteins quantified in at least 3 samples in proteomic analysis by mass spectrometry

S3 Data. Lists of proteins found to be significantly changed (≥1.5 fold-change, p-value ≤ 0.05) compared to growth-matched controls on Day 4, Day 6, and Day 7

S4 Data. Student's two-way t-tests for proteomic analysis for Day 4, Day 6, and Day 7

S5 Data. Metascape results for protein lists significantly changed (≥1.5 fold-change, p-value ≤ 0.05) compared to growth-matched controls on Day 4, Day 6, and Day 7

Figure S1

Target 1

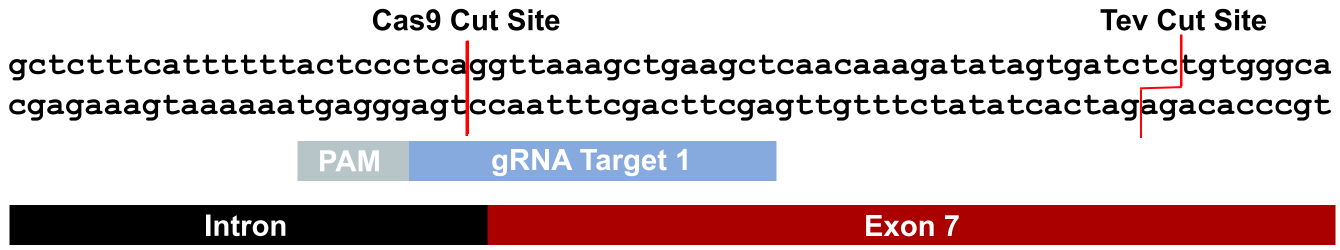

Target 2

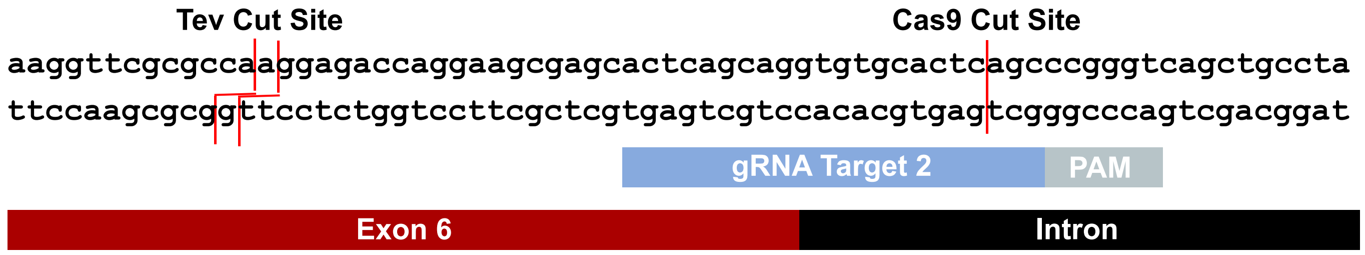

Target 3

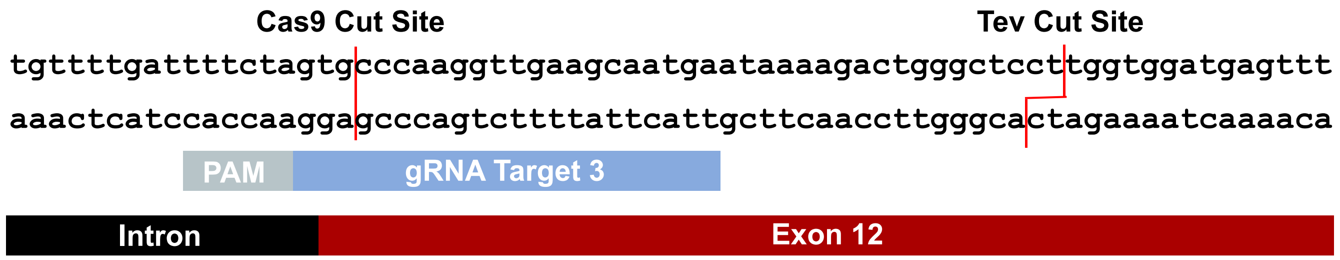

Figure S2

a

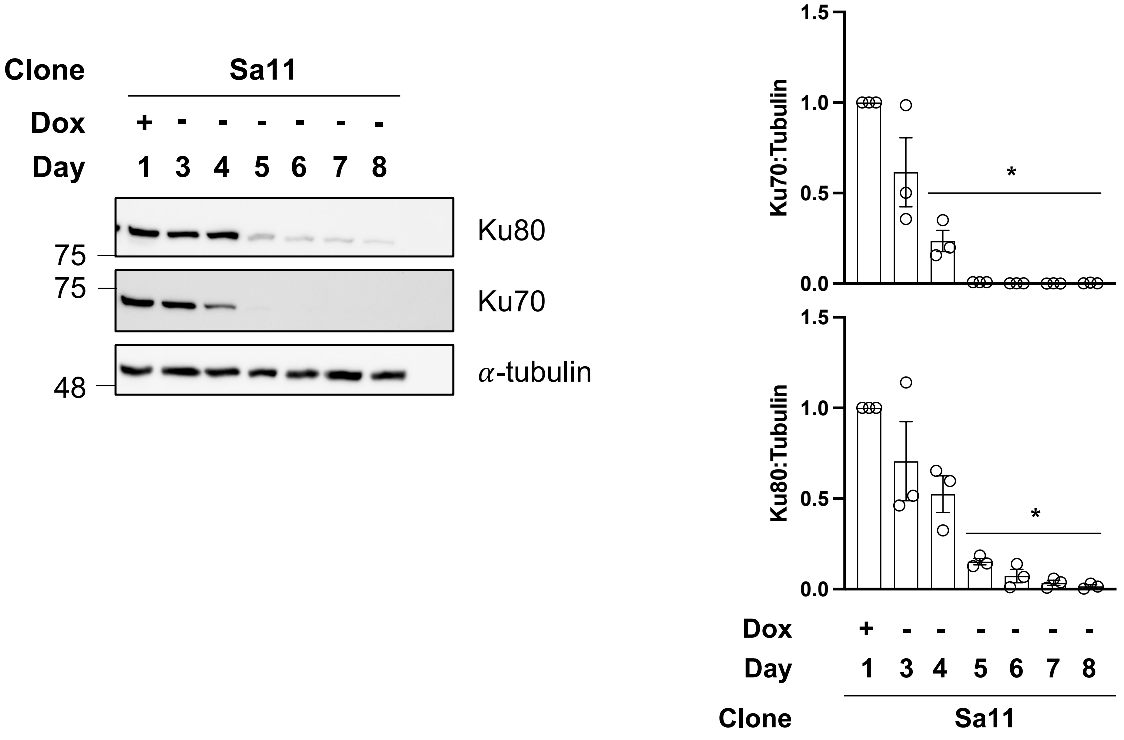
Clone
Sa11
Dox
+ - - - - - -
Day
1 3 4 5 6 7 8
Ku80
Ku70
α-tubulin
75
75
48
Ku70:Tubulin
Ku80:Tubulin
1.5
1.0
0.5
0.0
*

b

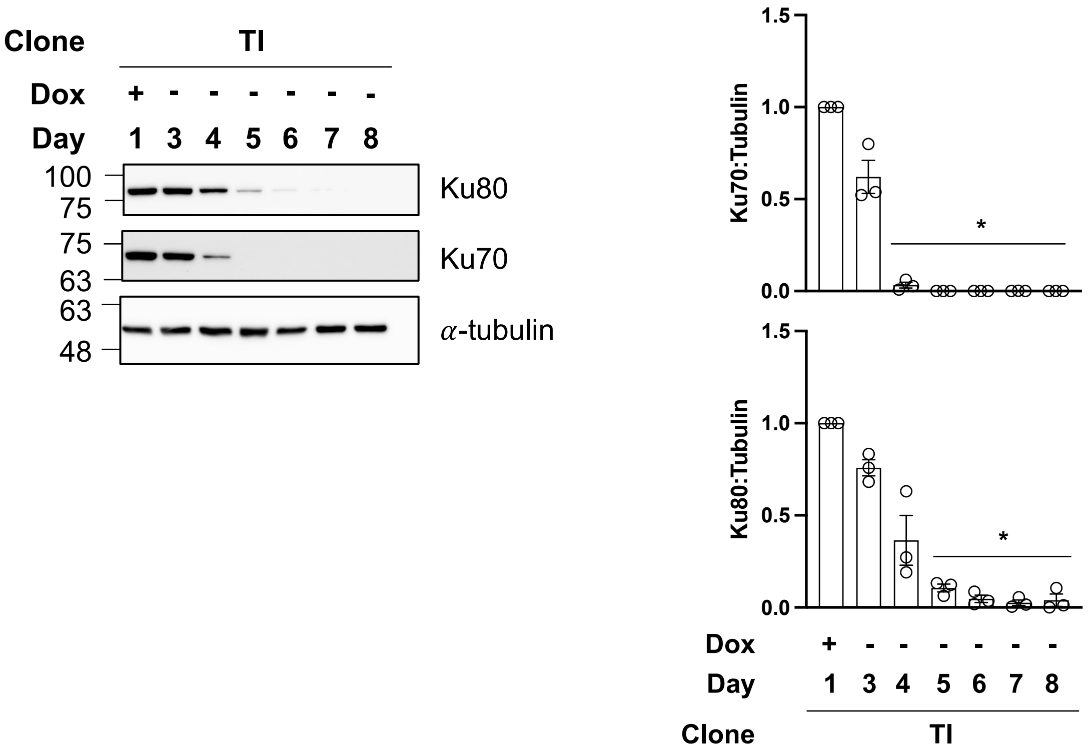
Clone
TI
Dox
+ - - - - - -
Day
1 3 4 5 6 7 8
Ku80
Ku70
α-tubulin
100
75
75
63
63
48
Ku70:Tubulin
Ku80:Tubulin
1.5
1.0
0.5
0.0
*

Figure S3

a

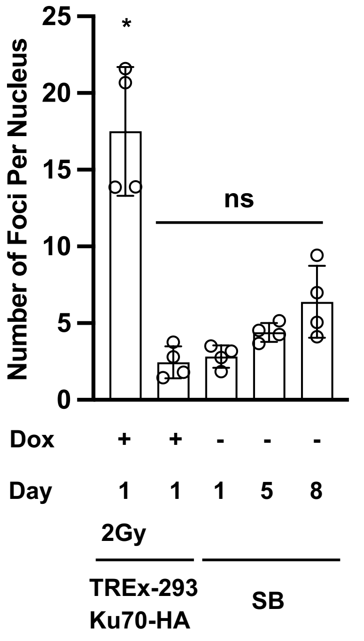

25
20
15
10
5
0
Number of Foci Per Nucleus
*
ns
Dox
+
+
-
-
-
Day
1
1
1
5
8
2Gy
TREx-293
Ku70-HA
SB

Figure S4

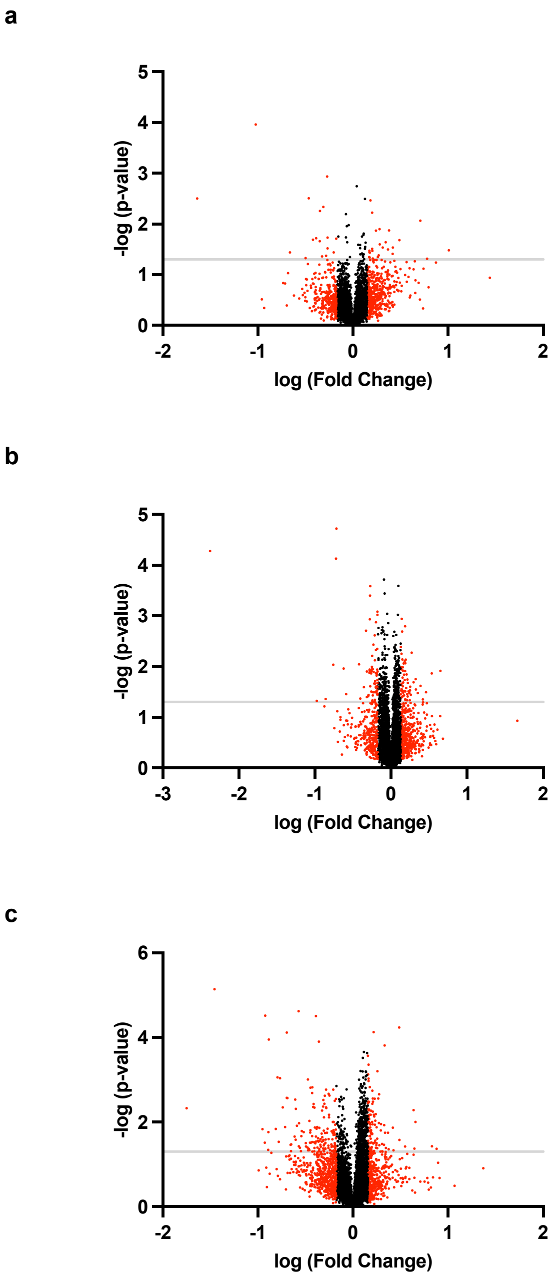
a
-log (p-value)
log (Fold Change)
b
-log (p-value)
log (Fold Change)
c
-log (p-value)
log (Fold Change)

Figure S5

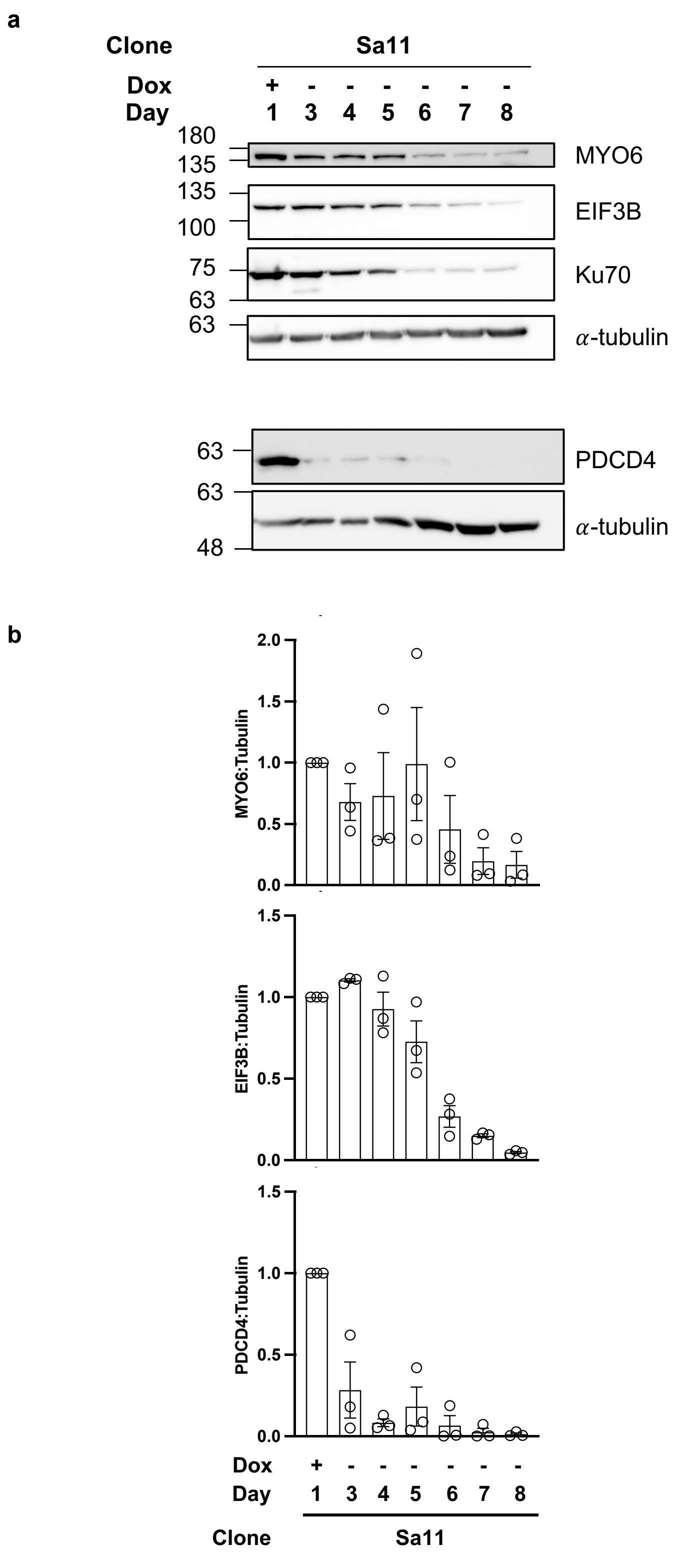
a
Clone
Sa11
Dox
+ - - - - - -
Day
1 3 4 5 6 7 8
180
135
MYO6
135
100
EIF3B
75
63
Ku70
63
α-tubulin
63
PDCD4
63
48
α-tubulin
b
MYO6:Tubulin
2.0
1.5
1.0
0.5
0.0
EIF3B:Tubulin
1.5
1.0
0.5
0.0
PDCD4:Tubulin
1.5
1.0
0.5
0.0
Dox
+ - - - - - -
Day
1 3 4 5 6 7 8
Clone
Sa11

S6 Figure

Fig 1 a

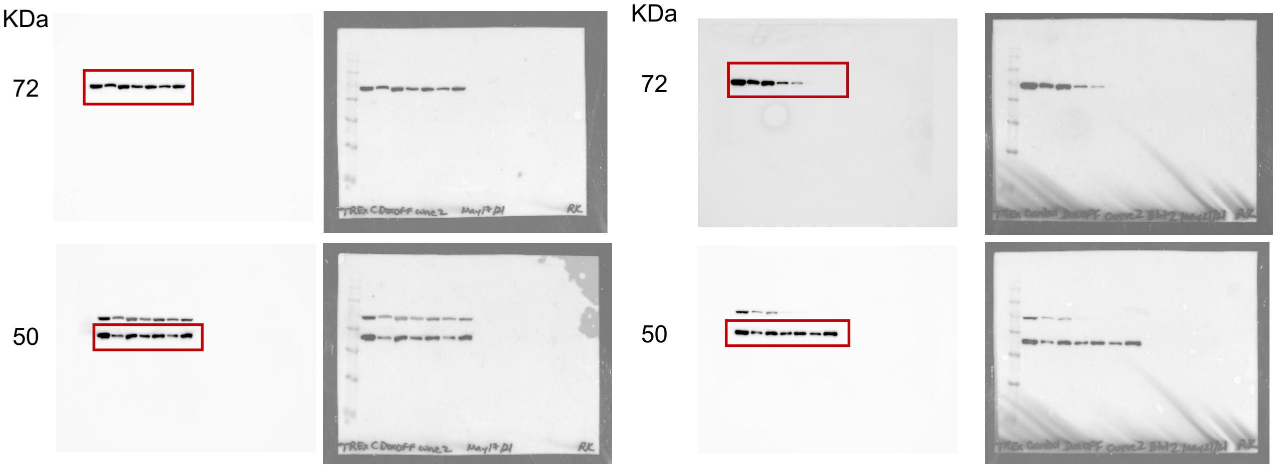

Antibody: Ku70 (Top Left), Tubulin (Bottom Left), HA (Top Right), Tubulin (Bottom Right). Merged images with ladder provided beside every uncropped chemiluminescent image.
SDS-PAGE 10% gels

Fig 1 d

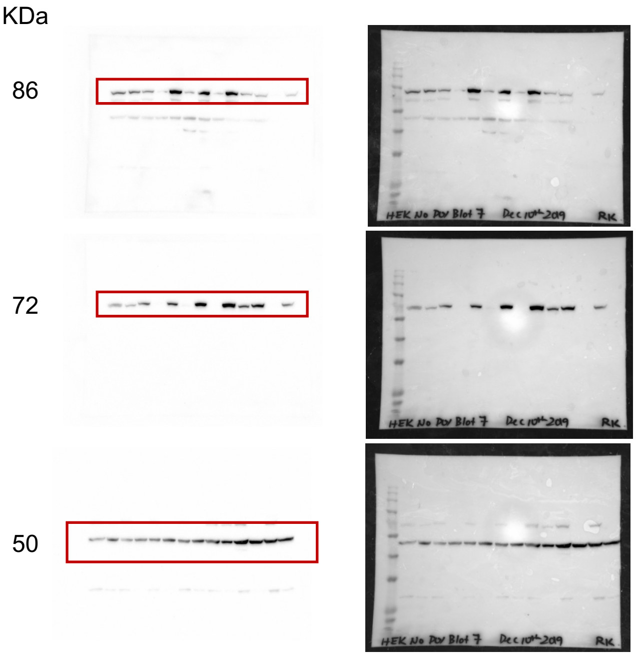

Antibody: Ku80 (Top), Ku70 (Middle), Tubulin (Bottom). Merged images with ladder provided beside every uncropped chemiluminescent image.
SDS-PAGE 10% gel

Fig 2 a

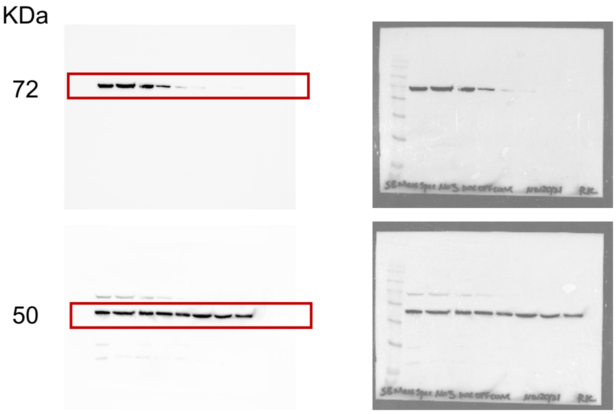

Antibody: Ku70 (Top), Tubulin (Bottom). Merged images with ladder provided beside every uncropped chemiluminescent image.
SDS-PAGE 10% gel

Figure S6 continued

Fig 4 c

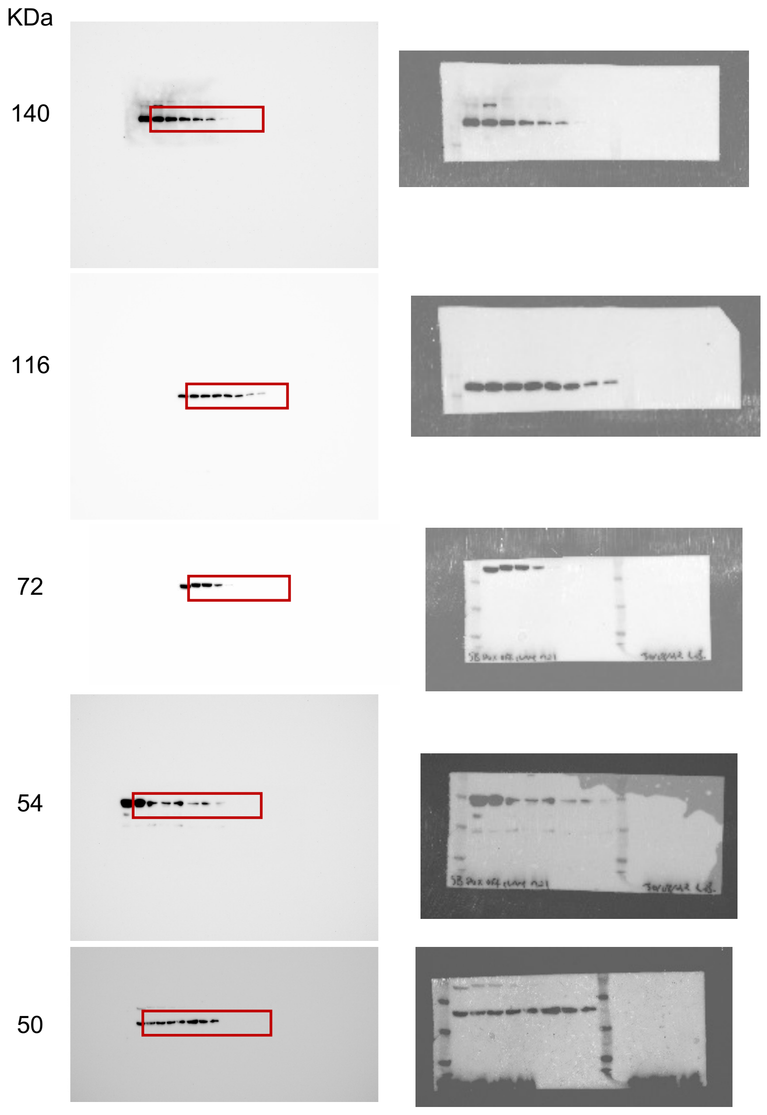

Antibody: Myosin VI (Top), EIF3η (2nd), Ku70 (3rd), PDCD4 (4th), Tubulin (Bottom). Merged images with ladder provided beside every uncropped chemiluminescent image.
SDS-PAGE 10% gel

Figure S6 continued

Fig S2 a

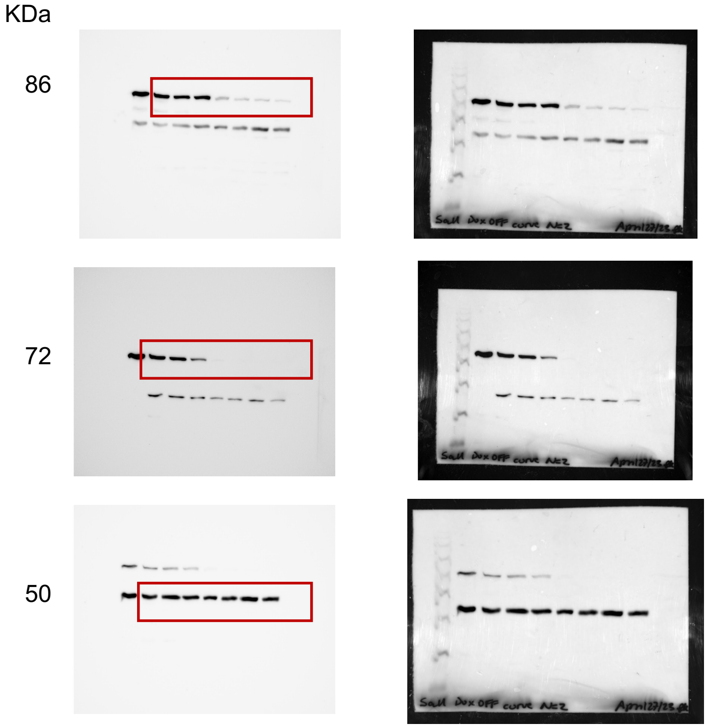

Antibody: Ku80 (Top), Ku70 (Middle), Tubulin (Bottom). Merged images with ladder provided beside every uncropped chemiluminescent image.
10% SDS PAGE gel

Fig S2 b

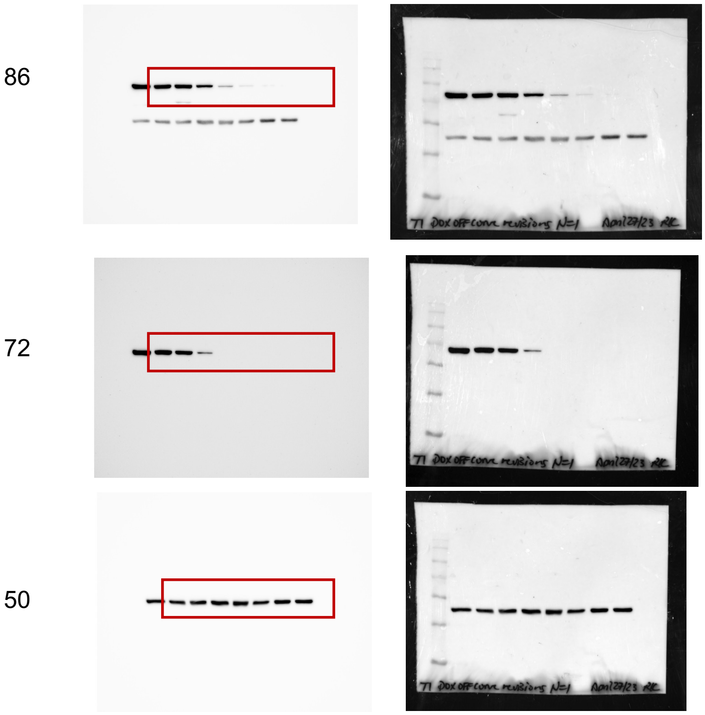

Antibody: Ku80 (Top), Ku70 (Middle), Tubulin (Bottom). Merged images with ladder provided beside every uncropped chemiluminescent image.
10% SDS PAGE gel

Figure S6 continued

Fig S5 a

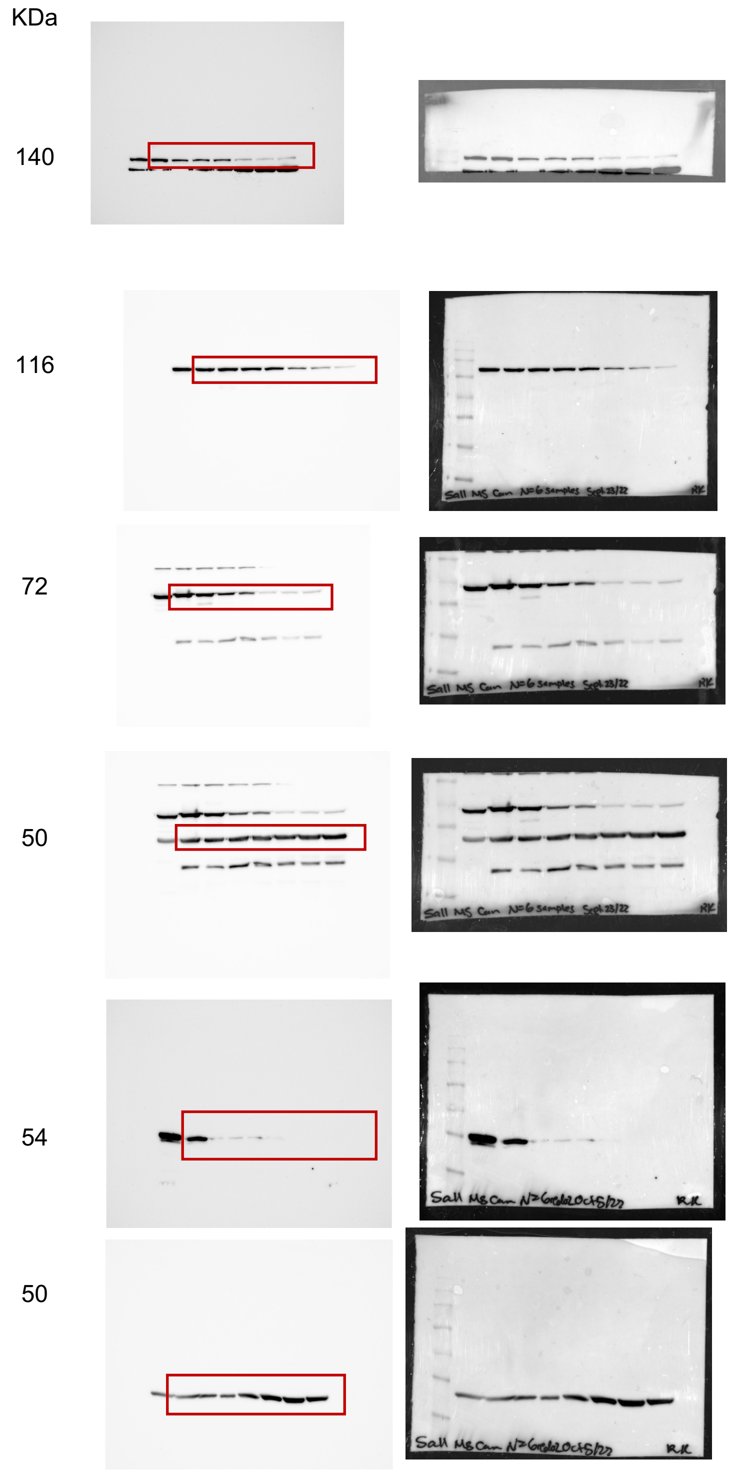

Antibody: Myosin VI (Top), EIF3η (2nd), Ku70 (3rd), Tubulin (4th), PDCD4* (5th), Tubulin* (Bottom). Merged images with ladder provided beside every uncropped chemiluminescent image. * Indicates different blot membrane. SDS-PAGE 10% gels

Figure S7

Fig 3 a

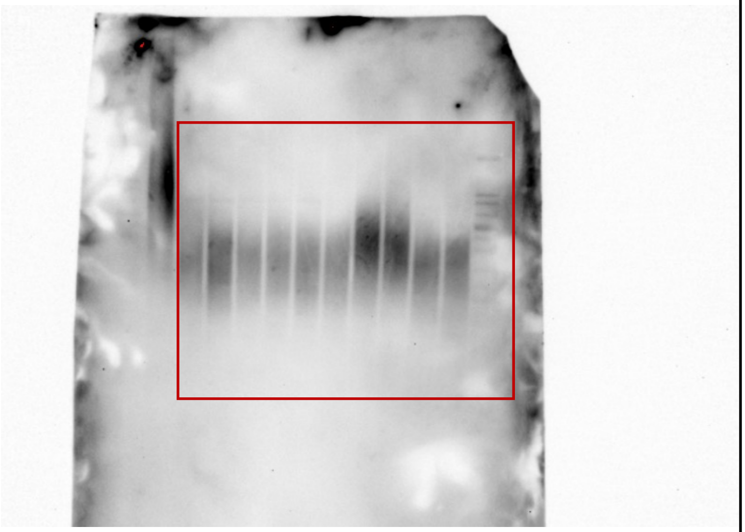

Southern Blot
Samples ran on a 0.8% agarose gel
